# Supplementary material for: Use of the Visceral Adiposity Index as an Indicator of Chronic Kidney Disease in Older Adults: Comparison with Body Mass Index
Source: J Clin Med. 2022 Oct 26;11(21):6297. doi: 10.3390/jcm11216297 (PMC9659218; doi:10.3390/jcm11216297)
Supplement: Supplementary file 1 [file jcm-11-06297-s001.zip › jcm-1944838-supplementary.pdf]

Table S1. Characteristics of study subjects and differences between males and females

	Overall (n = 7736)	Male (n = 3479)	Female (n = 4257)	*P* Value
Glucose, mg/dL	107.5 ± 26.0	107.6 ± 26.2	107.3 ± 25.8	= 0.611
Total cholesterol, mg/dL	187.0 ± 39.5	185.7 ± 39.6	188.0 ± 39.3	< 0.05
Handgrip strength, kg	24.6 ± 8.6	24.6 ± 8.7	24.5 ± 8.6	= 0.658
Total energy intake, kcal/d	1729.4 ± 729.2	1735.4 ± 735.8	1724.4 ± 723.9	= 0.509
Carbohydrate intake, g	293.5 ± 120.2	295.2 ± 120.7	292.2 ± 119.8	= 0.274
Protein intake, g	58.3 ± 30.3	58.3 ± 30.8	58.3 ± 29.9	= 0.945
Fat intake, g	29.3 ± 22.7	29.0 ± 23.1	29.5 ± 22.4	= 0.359
Medication (%)				
Dyslipidemia	1999 (25.8)	663 (8.6)	1336 (17.3)	< 0.001
Diabetes	1474 (19.1)	705 (9.1)	769 (9.9)	< 0.05
Hypertension	3741 (48.4)	1631 (21.1)	2110 (27.3)	< 0.05
Household income (%)				< 0.001
Low	2922 (37.8)	1124 (32.3)	1798 (42.2)	
Lower-middle	2244 (29.0)	1068 (30.7)	1176 (27.6)	
Upper middle	1455 (18.8)	696 (20.0)	759 (17.8)	
High	1115 (14.4)	591 (17.0)	524 (12.3)	
Education level (%)				< 0.001
Primary school	3849 (49.8)	1216 (35.0)	2633 (61.9)	
Middle school	1330 (17.2)	639 (18.4)	691 (16.2)	
High school	1578 (20.4)	945 (27.2)	633 (14.9)	
College	979 (12.7)	679 (19.5)	300 (7.0)	
Alcohol consumption (%)				= 0.104
Never	3385 (43.8)	1491 (42.9)	1894 (44.5)	
≤ once a week	2911 (37.6)	1299 (37.3)	1612 (37.9)	
2-3 times/week	809 (10.5)	385 (11.1)	424 (10.0)	
≥ 4 times/week	631 (8.2)	304 (8.7)	327 (7.7)	
Smoking (%)				< 0.01
Never	4717 (61.0)	2046 (58.8)	2671 (62.7)	
Former smoking	2207 (28.5)	1044 (30.0)	1163 (27.3)	
Current smoking	812 (10.5)	389 (11.2)	423 (9.9)	

Values are means ± SD. The independent t-test or Mann–Whitney U test were used to compare the males and females.

Table S2. Sex-specific differences and trends of subjects by eGFR category

	eGFR category (mL/min/1.73 m^2)			*P* for difference	*SS*‡	*P* for trend‡
	A	B	C			
Male, n	1752	1185	320			
Glucose, mg/dL	100.0 ± 22.0 (98.9, 101.0)	102.6 ± 22.9 (101.3, 103.9)	104.7 ± 25.9 (101.9, 107.5)	A < B, C	7.72	< 0.001
Cholesterol, mg/dL	191.2 ± 36.5 (190.4, 193.8)	195.2 ± 38.1 (193.0, 197.3)	191.7 ± 38.5 (187.5, 196.0)	NS	1.82	= 0.069
Handgrip, kg	28.2 ± 9.7 (27.8, 28.7)	28.9 ± 9.9 (28.3, 29.4)	27.7 ± 9.6 (26.7, 28.8)	NS	0.81	= 0.416
TEI, kcal/d†	2038.8 ± 914.1 (1984.3, 2070.6)	1972.1 ± 828.2 (1916.9, 2010.7)	1745.2 ± 818.6 (1704.5, 1873.8)	A, B > C	-3.84	< 0.05
Carbo, g	308.5 ± 135.5 (302.2, 314.9)	315.2 ± 127.6 (308.0, 322.5)	298.7 ± 115.2 (286.0, 311.3)	NS	0.80	= 0.427
Protein, g†	72.7 ± 42.2 (70.7, 74.6)	68.5 ± 37.4 (66.3, 70.6)	60.4 ± 34.9 (56.6, 64.3)	A > B > C	-5.35	< 0.001
Fat, g†	44.4 ± 34.7 (42.8, 46.0)	39.1 ± 30.3 (37.3, 40.8)	33.8 ± 31.1 (30.4, 37.2)	A > B > C	-7.38	< 0.001
Female, n	2048	1410	338			
Glucose, mg/dL	101.0 ± 24.7 (100.0, 102.1)	103.9 ± 24.9 (102.6, 105.2)	103.6 ± 25.0 (100.9, 106.3)	A < B	6.46	< 0.001
Cholesterol, mg/dL†	191.9 ± 35.5 (190.4, 193.4)	192.8 ± 39.0 (190.7, 194.8)	192.9 ± 42.0 (188.4, 197.4)	NS	0.43	= 0.669
Handgrip, kg	28.6 ± 9.5 (28.2, 29.9)	27.8 ± 10.0 (27.3, 28.3)	27.1 ± 9.5 (26.1, 28.1)	NS	-1.27	= 0.205
TEI, kcal/d†	2012.2 ± 901.5 (1973.1, 2051.3)	1901.8 ± 833.3 (1858.3, 1945.4)	1815.0 ± 768.0 (1732.8, 1897.2)	A > B, C	-1.81	= 0.071
Carbo, g	307.2 ± 132.6 (301.5, 312.9)	309.6 ± 131.0 (302.8, 316.4)	303.8 ± 119.6 (291.0, 316.6)	NS	-0.44	= 0.662
Protein, g†	70.9 ± 37.6 (69.3, 72.5)	64.8 ± 34.6 (63.0, 66.6)	61.6 ± 36.0 (57.8 65.5)	A > B > C	-6.12	< 0.001
Fat, g†	43.8 ± 33.3 (42.4, 45.2)	36.5 ± 28.0 (35.0, 38.0)	32.0 ± 24.7 (29.4, 34.7)	A > B > C	-8.88	< 0.001

Values are means ± SD (95% CI). †Mann-Whitney U test was applied to assess the difference between groups. ‡Jonckheere-Terpstra test was used to assess the trend among three groups. A = eGFR ≥ 60 (95% CI); B = eGFR 45-59.9 (95% CI); C = eGFR < 45 (95% CI); Carbo = Carbohydrate TEI = Total energy intake; *SS* = standardized statistic; NS = not significant
